# Otolaryngology Consult Protocols in the Setting of COVID-19: The University of Pittsburgh Approach

**DOI:** 10.1177/00034894211005937

**Published:** 2022-01

**Authors:** Harish Dharmarajan, Michael A. Belsky, Jennifer L. Anderson, Shaum Sridharan

**Affiliations:** 1 Department of Otolaryngology, University of Pittsburgh Medical Center, Pittsburgh, PA, USA; 2 University of Pittsburgh School of Medicine, University of Pittsburgh, Pittsburgh, PA, USA

**Keywords:** consult, COVID-19, protocol, safety, training

## Abstract

**Objective:**

To analyze trends in otolaryngology consultations and provide algorithms to guide management during the COVID-19 pandemic.

**Methods:**

A retrospective cohort study at a single institution tertiary care hospital. A total of 95 otolaryngology consultations were performed from March 1, 2020 to April 26, 2020 (COVID-era) and 363 were performed from September 1, 2019 to February 29, 2020 (pre-COVID-era) at the UPMC Oakland campus. Data collected included patient demographics, COVID-19 status, reason for consult, location of consult, type of consult, procedures performed, need for surgical intervention, length of hospital stay and recommended follow up.

**Results:**

Patient populations in the pre-COVID-era and COVID-era were similar in terms of their distribution of demographics and chief complaints. Craniofacial trauma was the most common reason for consultation in both periods, followed by vocal fold and airway-related consults. We saw a 21.5% decrease in the rate of consults seen per month during the COVID-era compared to the 6 months prior. Review of trends in the consult workflow allowed for development of several algorithms to safely approach otolaryngology consults during the COVID-19 pandemic.

**Conclusions:**

Otolaryngology consultations provide valuable services to inpatients and patients in the emergency department ranging from evaluation of routine symptoms to critical airways. Systematic otolaryngology consult service modifications are required in order to reduce risk of exposure to healthcare providers while providing comprehensive patient care.

## Introduction

As coronavirus disease 2019 (COVID-19), caused by severe acute respiratory syndrome coronavirus 2 (SARS-CoV-2), continues to spread across the globe, healthcare systems have now gathered significant amounts of data to assess the far-reaching consequences of this pandemic. Across medical specialties, healthcare professionals have radically modified daily routines and procedures to protect the health and safety of all patients as well as healthcare workers.

High viral loads of SARS-CoV-2 have been detected in the upper respiratory tracts of infected patients. Patients diagnosed with COVID-19 infection demonstrated a higher viral load soon after symptom onset, with higher viral loads detected in the nose than the throat.^1^ Furthermore, viral loads detected in an asymptomatic patient may be similar to those of symptomatic patients. An additional cohort study of 23 patients diagnosed with COVID-19 in Hong Kong demonstrated posterior oropharyngeal salivary viral load to peak in the first week after symptom onset and subsequently decline over time; however, viral RNA was still detected in posterior oropharyngeal saliva at least 20 days after symptom onset in one-third of patients.^2^ These findings support the need for the otolaryngology community to modify patient care delivery during the COVID-19 pandemic.

Due to the nature of the head and neck examination and procedures, otolaryngologists have been vigilant in their response to the pandemic. Endoscopy in particular is considered to be a high-risk procedure due to potential for aerosolization. It is unclear how long aerosolized particles remain airborne post-endoscopy, though some reports suggest particles may remain airborne for up to 3 hours.^3^ This results in risk not only to otolaryngology providers but to members of additional care teams. Many groups have published safety guidelines and recommendations for high-risk procedures in a galvanizing effort to share helpful information.^4-12^

Handling otolaryngology consultations during the COVID-19 pandemic presents a unique challenge for providers. The care team encounters a new patient, possibly with unknown COVID-19 status, and must perform all diagnostic and clinical decision-making while balancing the risks of exposure. Algorithms for management of common pediatric otolaryngology consults have been proposed previously.^13^ Similar management algorithms for adult otolaryngology consults have yet to be established. The purpose of this study is 2-fold: first, to compare otolaryngology consult service data during the onset of the COVID-19 pandemic to those of the previous 6 months at our institution, and second, to establish algorithms for safely approaching consults during the pandemic.

## Methods

We performed a retrospective chart review of patients seen by the consultation service of the Department of Otolaryngology at the University of Pittsburgh Medical Center (UPMC), a tertiary care center. Data collection was approved by our institutional review board (IRB#STUDY20040289). Patients included in the study were seen as consults between March 1, 2020 and April 26, 2020, either in the emergency department (ED) or inpatient wards. Our consult service maintained an ongoing list of all patients seen in this time period; data was collected via direct chart review. Collected data included basic demographic and clinical information such as reason for consult, chief complaint, inpatient interventions and duration of hospitalization. For determination of hospitalization duration, if a patient was still admitted at the time of data collection, we considered the date of data acquisition to be their “discharge” date. For patients who died during admission, we considered their date of death to be their “discharge” date.

The COVID-19 status at the time of initial consultation was recorded; patients were COVID-positive, under investigation, COVID-negative, or with an unknown status but negative screening assessment. At our institution, patients are “under investigation” if they present with signs and symptoms suspicious for SARS-CoV-2 infection. Patients with “unknown” status do not have symptoms suggestive of COVID-19 (screened negative), and thus were treated with similar precautions as those with known negative status, though approaches remain guarded due to acknowledgement of potential asymptomatic carriers of SARS-CoV-2.^14^ For comparison, we grouped patients under investigation and COVID-positive patients together, while COVID-negative and unknown status with negative screen patients formed a second group.

We also analyzed consult data in the pre-COVID era from September 1, 2019 through February 29, 2020 to establish a baseline for the consult service workflow. Data were obtained via financial billing records. We used International Classification of Disease, 10th edition (ICD-10) codes and descriptions to group patients into chief complaint categories. We used Common Procedural Technology (CPT) codes to study which procedures these patients underwent during their hospitalizations.

A Wilcoxon rank sum test was used to determine whether patient ages were significantly different in the 2 time periods, as ages were not normally distributed. Chi-square tests were utilized to determine if there were significant differences in the distributions of gender, chief complaints, proportion of patients requiring rigid or flexible endoscopy, and proportion of patients requiring operative interventions.

After review of changing consult patterns and forthcoming literature on safe patient care during the pandemic, we developed several algorithms for approaching the most common consults at our institution. These algorithms were developed by iterative discussion among attending physicians and house staff with consideration of evolving literature on the topic.

## Results

Between March 1, 2020 and April 26, 2020, the otolaryngology service was consulted for 95 patients. The clinical characteristics of these patients are displayed in Table 1. Notably, only 8 patients were under investigation for SARS-CoV-2 infection at the time of initial evaluation and only 2 patients had tested positive. Eleven patients were COVID-19-negative and the remaining 74 had unknown infection status but negative screens. The median age of all patients was 55 years with roughly similar age, gender, and chief complaint distributions between patients with positive/under-investigation COVID-19 status and negative status/negative screen patients. The most common reason for consult was craniofacial trauma, followed by vocal fold/airway evaluations and epistaxis. Consults in the “other” designation consisted of skull base surgery-related consults (n = 5), skull base osteomyelitis (n = 1), nasal foreign body (n = 1), dysphagia (n = 1), feeding tube placement (n = 1), and abnormal head and neck exam (n = 1). Similar percentages of patients required bedside procedures or operative interventions. Operative procedures performed by the consult team included tracheostomy placement (n = 3), incision and drainage of head and neck abscess (n = 1), control of post-operative bleeding (n = 1), facial trauma repair (n = 3), incisional biopsy of a neck mass (n = 1), head and neck malignancy resection with reconstruction (n = 1), and a tracheocutaneous fistula repair (n = 1). When possible, consults were completed via telemedicine to protect providers from potential exposure; only 16% of consults could be completed virtually.

**Table 1.** Consults in the COVID-19 Era (3/1/20-4/26/20).

	COVID+ (n = 10)	COVID− (n = 85)	Total (n = 95)
Age (years)	56.5 (19-93)	55 (18-93)	55 (18-93)
Gender			
Female	2 (20%)	32 (38%)	34 (36%)
Male	8 (80%)	53 (62%)	61 (64%)
Reason for consult/chief complaint			
Epistaxis	2 (20%)	10 (12%)	12 (13%)
Vocal fold or airway evaluation	1 (10%)	17 (20%)	18 (19%)
Tracheostomy-related	1 (10%)	7 (8%)	8 (8%)
Head and neck mass	0 (0%)	8 (9%)	8 (8%)
Head and neck infection	1 (10%)	6 (7%)	7 (7%)
Otologic complaint	0 (0%)	9 (11%)	9 (10%)
Craniofacial trauma	3 (30%)	20 (24%)	23 (24%)
Other	2 (20%)	8 (9%)	10 (11%)
Consult location			
Inpatient	8 (80%)	49 (58%)	57 (60%)
ED	2 (20%)	36 (42%)	38 (40%)
Patients admitted from ED	2 (20%)	24 (28%)	26 (27%)
Type of consult			
In-person	8 (80%)	72 (85%)	80 (84%)
E-consult	2 (20%)	13 (15%)	15 (16%)
Patients requiring bedside procedure	5 (50%)	34 (40%)	39 (41%)
Rigid or flexible endoscopy	3 (30%)	16 (19%)	19 (20%)
Nasal manipulation	5 (50%)	26 (31%)	31 (33%)
Oral manipulation	2 (20%)	18 (21%)	20 (21%)
Lower respiratory tract manipulation	1 (10%)	6 (7%)	7 (7%)
Operative intervention required	2 (20%)	9 (11%)	11 (12%)
Duration of hospital stay (days)	8 (2-72)	5 (1-467)	6 (1-467)
Recommended follow-up time (weeks)	2.5 (1-4)	2 (1-16)	2 (1-16)
Follow-up if needed	0 (0%)	9 (11%)	9 (9%)
Follow-up unnecessary	2 (20%)	20 (24%)	22 (23%)

*Note.* COVID+ are patients under investigation and patients tested positive. COVID− are patients with unknown status (negative screen) and those who tested negative. Continuous outcomes reported as median (range). Categorical outcomes reported as count (%)

Abbreviation: ED, emergency department.

In the 6 months prior to March 2020, the consult service evaluated 363 patients (Table 2). The distribution of consult chief complaints was similar to that in the COVID-era, with craniofacial trauma being the most common followed by the “other” category and vocal fold/airway-related symptoms. For this group, the “other” category consisted of a wider variety of chief complaints for consults, with the most frequent reasons including rhinologic symptoms/chronic sinusitis (n = 5), skull base-surgery-related presentations (n = 4), dysphagia (n = 4), and post-operative care or complications (n = 5). Twice as many patients had operative interventions per month in the pre-COVID era (11.5 patients per month compared to 5.5 patients per month). Similar proportions of patients underwent rigid and flexible endoscopy (17% in previous 6 months vs 20% in COVID-19 era). There were no statistically significant differences in age (*P* = .839), gender (*P* = .553), consult chief complaints (*P* = .450), proportion of patients requiring rigid or flexible endoscopy (*P* = .465), or proportion of patients requiring an operative intervention (*P* = .090) between the pre-COVID era patient group and the COVID-era group. Of the 74 patients who underwent rigid or flexible endoscopy, 63 underwent flexible fiberoptic laryngoscopy and 11 underwent rigid nasal endoscopy.

**Table 2.** Consult Comparison: Pre-COVID versus COVID-Era.

	Pre-COVID (n = 363)	COVID Era (n = 95)	*P*-value
Age (years)	54.4 (19.0)	55.7 (18.1)	.839
Gender			.553
Female	142 (39%)	34 (36%)	
Male	221 (61%)	61 (64%)	
Reason for consult/chief complaint			.450
Epistaxis	34 (9%)	12 (13%)	
Vocal fold or airway evaluation	61 (17%)	18 (19%)	
Tracheostomy-related	18 (5%)	8 (8%)	
Head and neck mass	17 (5%)	8 (8%)	
Head and neck infection	35 (10%)	7 (7%)	
Otologic complaint	29 (8%)	9 (10%)	
Craniofacial trauma	100 (27%)	23 (24%)	
Other	69 (19%)	10 (11%)	
Operative intervention required	69 (19%)	11 (12%)	.090
Underwent rigid or flexible endoscopy	74 (20%)	19 (20%)	.465

*Note.* Age presented as mean (SD). All categorical data presented as count (%).

Monthly rates of consults for epistaxis, vocal fold or airway evaluation, tracheostomy-related care, head and neck mass, head and neck infections, and otologic complaints were similar, but consults related to craniofacial trauma or other chief complaints decreased in the COVID era. While the overall consult rate dropped by 21.5%, the rate of craniofacial trauma consults decreased by 31.1% and the rate of “other” consults decreased by 47.8%. The monthly rate of consults requiring operative procedures decreased by 52.2%, and the monthly number of consult patients undergoing rigid or flexible endoscopy decreased by 20.8% (Table 3).

**Table 3.** Rates of Consults in Pre-COVID versus COVID Era.

	Pre-COVID (n = 363)	COVID Era (n = 95)
Consults per month	60.5	47.5
Epistaxis	5.7	6
Vocal fold or airway evaluation	10.2	9
Tracheostomy-related	3	4
Head and neck mass	2.8	4
Head and neck infection	5.8	3.5
Otologic complaint	4.8	4.5
Craniofacial trauma	16.7	11.5
Other	11.5	5
Requiring operative intervention	11.5	5.5
Underwent rigid or flexible endoscopy	12.3	9.5

*Note.* Monthly data are presented as mean number of patients per month.

## Discussion

Although the city of Pittsburgh and its surrounding communities have had a relatively low number of confirmed cases of COVID-19 compared to other parts of the United States, our institution and department enacted significant measures to protect healthcare workers and prevent the spread of the virus in our hospitals.

The UPMC Department of Otolaryngology provides consultation to 7 hospitals. Prior to COVID-19, resident consultation coverage included multiple hospitals. During the COVID-19 area, resident coverage was limited to only 1 hospital. Self-contained teams of residents and attendings were formed to minimize cross-contamination between teams in case of potential exposure. Throughout our hospital system, special precautions are taken during each patient encounter based on patients’ COVID status. Telehealth and e-consult measures are encouraged if applicable, and increased attention is given to the disinfection of commonly used otolaryngology instruments. Understanding how otolaryngology consult approaches have changed in the COVID-19 era is vital for strategizing safe and effective means to deliver patient care and collaborate with other healthcare providers. While the relative proportions of chief complaints for consults have not changed drastically, comparisons of monthly rates suggest important differences in patient management/exposure in the COVID-19 era. The volume of patients requiring operative intervention decreased by more than 50%, and a narrower range of procedures are being performed in the operating room (OR). Providers in other fields have noted decreased admissions for various conditions; De Filippo et al. found a significantly lower rate of acute coronary syndrome (ACS) related hospitalizations during the pandemic compared to ACS-related hospitalizations in the previous year and earlier in 2020.^15^ Decrease in craniofacial trauma consults at our institution is likely a reflection of social distancing measures and reduced participation in activities that predispose to traumatic injuries.

By assessing trends in consult workflow during the COVID-era, several algorithms were developed for safe, effective approaches to the most commonly encountered consults.

### Institutional Protocols for Common Consults

#### Epistaxis (Figure 1)

Due to the risk of aerosol transmission during epistaxis management, an updated set of recommendations has been published.^16,17^ All patients seen either in the emergency department (ED) or on the inpatient floors are treated as potentially COVID-19 positive. Full personal protective equipment (PPE) includes an N95 mask and face shield or surgical goggles. Relevant history should be obtained either from other providers or with appropriate contact precautions, and pertinent laboratory values are reviewed.

**Figure 1.**
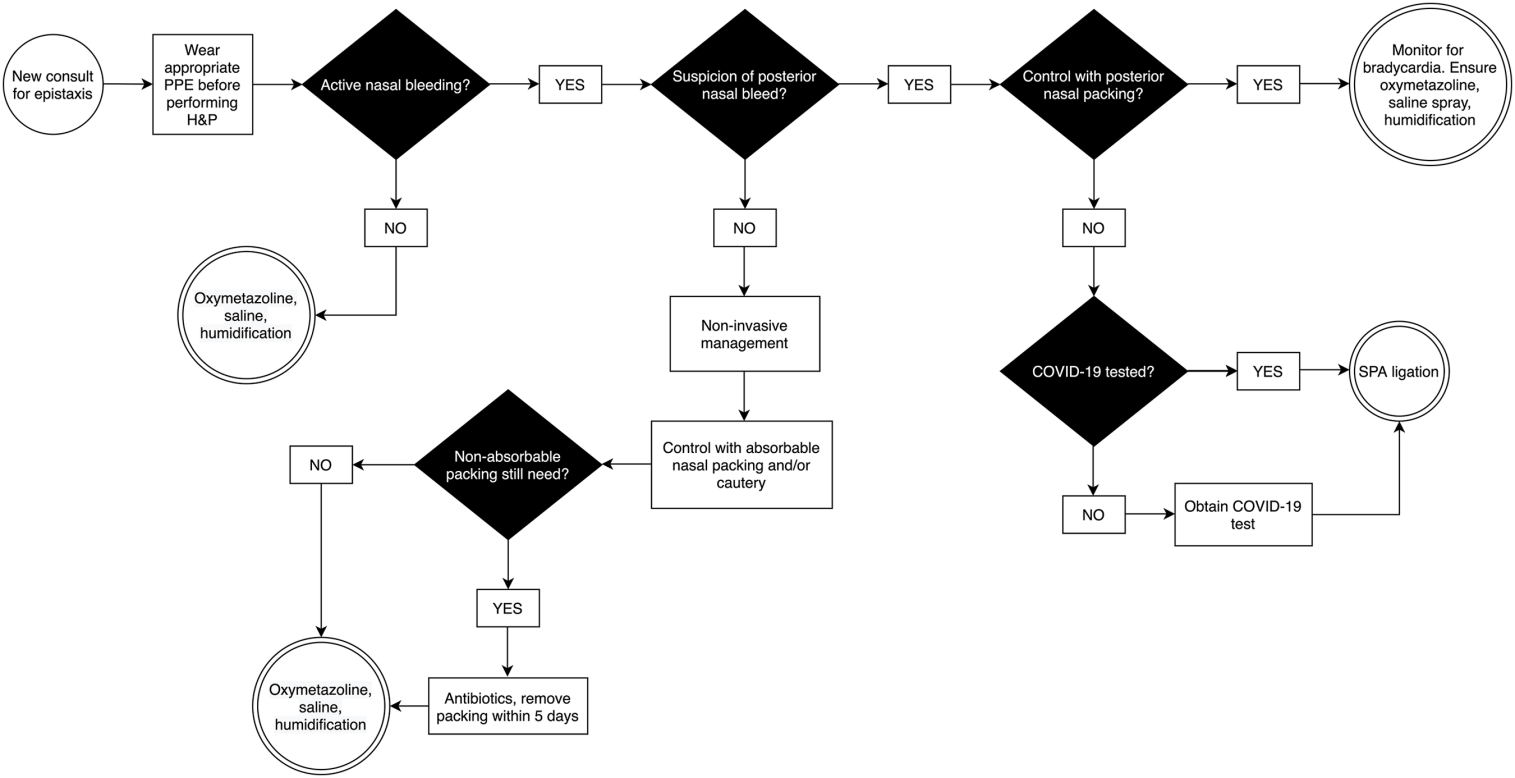
Algorithm for management of epistaxis. Abbreviations: PPE, personal protective equipment; H&P, history and physical exam; SPA, sphenopalatine artery.

Epistaxis management often requires manipulation of the nasal cavity for examination and achieving hemostasis. To minimize droplet aerosolization, non-invasive management including bidigital compression for at least 15 minutes and administration of antifibrinolytic agents such as tranexamic acid should be attempted first.^17^ This can be performed by emergency medicine providers, internists, or intensivists prior to otolaryngologist evaluation. Atomized sprays should be avoided; instead, soaked pledgets or cotton should be utilized for hemostasis and topical anesthesia. Anterior rhinoscopy using a headlight and nasal speculum is performed while wearing full PPE as described above. Rigid nasal endoscopy is deferred unless there is either a suspicion for an active posterior source or for evaluation of persistent, recurrent epistaxis, raising suspicion for an underlying sinonasal or nasopharyngeal mass. If non-invasive management fails, nasal packing or cautery should be attempted. Sphenopalatine artery (SPA) ligation is considered for suspected posterior bleeds only if bedside posterior packing is not sufficient; during the COVID-era, there has been a higher threshold for operative diagnostic nasal endoscopy and SPA ligation, and there has been a dedicated attempt to control all epistaxis cases at the bedside if feasible.

Absorbable packing is preferred to prevent an additional encounter for packing removal. Patients discharged from the ED with non-absorbable packing are instructed to return to clinic in approximately 5 days for packing removal. For inpatients, the primary team is asked to assist with packing removal in order to reduce patient encounters. After hemostasis is achieved, patients are instructed to complete a 3-day course of oxymetazoline twice daily and maintain intranasal humidification via saline spray, saline gel and home humidifier.

#### Airway evaluation (Figure 2)

Airway evaluations range from routine vocal fold evaluations to emergent endoscopies in patients with acute respiratory distress. Flexible fiberoptic laryngoscopy (FFL) is considered the gold standard for evaluation of the larynx and pharynx. However, FFL requires instrumentation of both the nasopharynx and oropharynx, resulting in potential aerosolization and transmission of viral particles. To address this risk of viral transmission during FFL, members of the American laryngology community provided a set of recommendations for the COVID-19 pandemic.^10^

**Figure 2.**
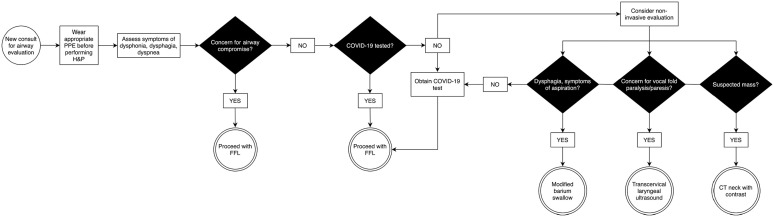
Algorithm for management of airway evaluation. Abbreviations: PPE, personal protective equipment; H&P, history and physical exam; FFL, flexible fiberoptic laryngoscopy.

FFL was recommended only to be performed in critical cases where findings have an immediate impact on patient management. Examples include hemoptysis and airway compromise due to infectious or malignant etiologies. For stable patients, alternative methods such as ultrasound or computed tomography (CT) imaging may be utilized. Ultrasound provides a quick method to evaluate lymphadenopathy, abscesses, neck masses, and vocal fold motion. Transcervical laryngeal ultrasonography provides a safer, less invasive alterative to evaluation of vocal fold motion.^18^

At our institution, the decision of whether FFL is necessary is shared by the most senior members of the consult team. Patients with unknown COVID-19 status are screened for symptoms concerning for possible infection. Despite absence of symptoms or negative COVID-19 testing, all patients are treated as potentially COVID-19 positive given the risk of false negative results and asymptomatic carriers. Examinations are performed with full PPE with senior team members present to reduce need for repeat endoscopy. Anesthetic gels are preferred instead of atomized anesthetics to reduce viral aerosolization risk. In cases where FFL is not crucial for patient management, alternative methods such as ultrasound and CT are utilized to assist in clinical decisions.

#### Tracheostomy-related care (Figure 3)

Tracheostomy placement and tracheostomy-related care are services regularly performed by otolaryngologists. Tracheostomy has previously demonstrated benefits including decreased sedation, lower risk of ventilator-associated pneumonia, shorter ICU stay, and shorter duration of mechanical ventilation,^19^ and prolonged intubation is associated with higher risk of subglottic and posterior glottic stenosis. However, early tracheostomy has not been demonstrated to significantly reduce post-intubation laryngotracheal stenosis.^19,20^

**Figure 3.**
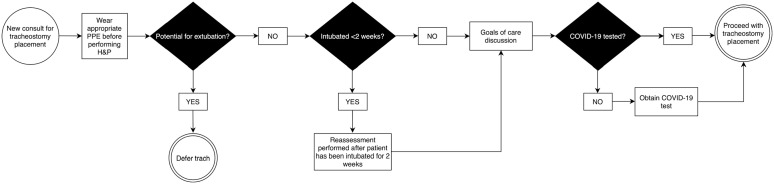
Algorithm for management of tracheostomy. Abbreviations: PPE, personal protective equipment; H&P, history and physical exam.

Determination of whether to perform a tracheostomy must consider patient prognosis as well as risks and benefits to the patient and providers. Goals of care discussions with patient family members are important regardless of COVID-19 status. Particularly in COVID-positive patients, where early reports demonstrated mortality rates exceeding 80% for patients requiring invasive mechanical ventilation,^21,22^ a discussion regarding the utility of tracheostomy is warranted. Our institution has modified our tracheostomy algorithm based on updated recommendations during the COVID-19 pandemic published by the Airway and Swallowing Committee of the American Academy of Otolaryngology-Head and Neck Surgery.^23^Additionally, our institution has changed our policy to only perform open tracheostomies. Other institutions have temporarily deferred performing percutaneous tracheostomies to decrease droplet aerosolization. If possible, tracheostomy placement is deferred until at least 2 weeks of intubation in order to allow for potential decrease in viral load, decreased risk of infection to providers and to provide greater opportunity for extubation. At least one COVID-positive patient for which we were consulted for tracheostomy was successfully extubated after delaying the procedure. Based on institution preference and location availability, tracheostomy may be performed in the OR or in a negative pressure room. COVID-19 testing is required prior to tracheostomy. Open tracheostomy within a negative pressure room is preferred to reduce risk of contamination during transport to and from the OR. A tracheostomy specific timeout is encouraged at the start of the case in collaboration with anesthesia and OR staff.^24^

Once the tracheostomy is performed, further adaptations are made to reduce potential viral transmission. Since the presence of a tracheostomy or laryngectomy stoma may pose increased risk of droplet and aerosol spread, guidelines have been proposed for tracheostomy management during the COVID-19 pandemic.^7,8^ Multiple methods are available to convert open airways to closed systems. Simple measures include partial closure with a Passy-Muir valve or tracheostomy cap, which are often employed for patients with an established tracheostomy, though these may not be tolerated in patients in the immediate post-operative setting. Heat moisture exchangers can also provide a droplet barrier in patients with tracheostomy or laryngectomy. Additionally, in-line suctioning systems can be employed to reduce risk of aerosolization during tracheostomy suctioning.

In patients who require tracheostomy tube change to an uncuffed tube to allow for use of Passy-Muir valve or transition toward decannulation, the appropriate PPE including N95 mask and face shield or goggles are utilized. Correct tracheostomy tube positioning is confirmed with visualization using a flexible fiberoptic endoscope, taking caution not to pass the distal end of the tube and manipulate the tracheal mucosa. Where once we would perform routine tracheostomy care including downsizing, we recommend deferring routine tracheostomy tube changes unless a different tube size or type is required for improving ventilation. Our decannulation protocol remains the same with the requirement that the provider assess both the upper airway and the airway distal to the tracheostomy tube to ensure patency before decannulation.

#### Head and neck mass (Figure 4)

When approaching a new consult for a head and neck mass, the consult resident should first obtain airway and COVID-19 status from the referring provider. Evaluation for underlying airway compromise is required, which is especially pertinent for extensive laryngeal malignancies where airway status may be tenuous, requiring an awake tracheostomy. If there is any concern for airway compromise, the patient must be evaluated promptly with full PPE in anticipation of performing bedside FFL. A brief history and physical exam in combination with CT imaging should provide a reference point for the expected level(s) of airway obstruction. FFL is then performed and recorded at the bedside. If proceeding with tracheostomy is necessary, timing of the case must be considered. If there is significant concern for rapid decompensation, an emergent awake tracheostomy must be performed. However, if symptoms are gradual and the patient’s airway status is stable, a tracheostomy can be done in a non-urgent manner. In either case, the on-call resident should do the following: (1) request a negative pressure OR room and ensure PPE availability, (2) confirm COVID-19 status and request testing if not yet performed, and (3) discuss the airway plan with the anesthesiologist. Once the tracheostomy is finished, direct laryngoscopy and biopsy should be performed at the end of the case.

**Figure 4.**
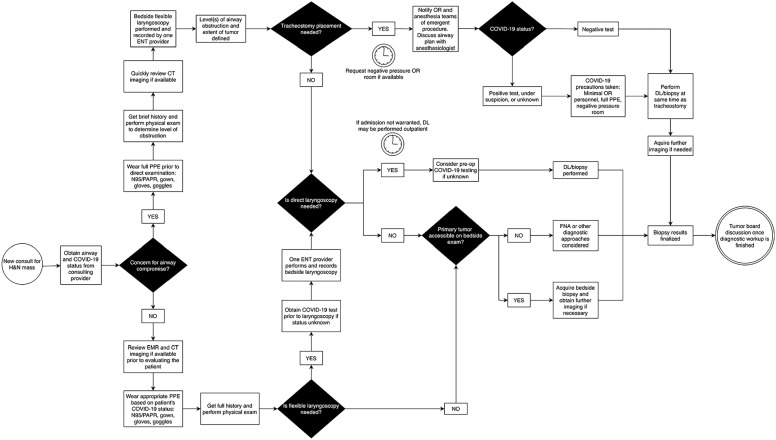
Algorithm for management of head and neck mass. Abbreviations: EMR, electronic medical record; PAPR, powered air purifying respirator; OR, operating room; DL, direct laryngoscopy; FNA, fine needle aspiration; CT, computed tomography.

If there is no concern for airway compromise on initial evaluation, the house-staff must determine first whether FFL is warranted. For malignancies based at the skin or oral cavity, this can be deferred unless there are symptoms suggesting a second primary. Biopsy should be performed at bedside and a definite plan for outpatient follow-up/intervention should be confirmed before discharge. A provider may consider fine needle aspiration of a neck mass rather than biopsy of an obvious primary site especially when a direct laryngoscopy is needed regardless: This strategy avoids manipulation of the upper aerodigestive tract at the bedside. For those with suspected oropharyngeal or laryngeal based malignancies, FFL should be performed and recorded at the bedside. If the patient requires direct laryngoscopy with biopsy for further evaluation, one must determine if it should be performed as an inpatient or if it may be scheduled as an outpatient. Given the current financial crisis, socioeconomic status of some patients, and transportation impediments during the COVID-19 pandemic, there may be greater risk of loss to follow-up or delay in care. If a patient under suspicion for new head and neck cancer does not warrant inpatient admission, there must be careful review of the risks for encouraging close outpatient follow-up. We recommend that pre-operative COVID-19 testing be completed before direct laryngoscopy is performed.

#### Head and neck infection (Figure 5)

As with the approach to a new head and neck mass, one should first evaluate for any airway compromise. If there is concern for airway involvement, FFL should be performed at the bedside. Available imaging is reviewed quickly, and the extent of the infection and level(s) of airway obstruction are defined. After evaluating for airway compromise, one should determine if there is an underlying abscess based on exam and imaging. If there is no abscess, the patient should start an empiric trial of antibiotics, commonly ampicillin-sulbactam, unless there is concern for a local infectious complication such as osteomyelitis, in which case an infectious disease consultation and prolonged antibiotic course may be required. If the abscess is accessible, a bedside incision and drainage should be performed with full PPE, limiting additional exposure to OR personnel. If the abscess is complicated (ie, with loculations or spanning multiple compartments) or difficult to access, operative drainage is required. When this is determined, the on-call house-staff should request COVID-19 testing and carefully assess whether any additional surgery teams are needed in order to limit OR personnel.

**Figure 5.**
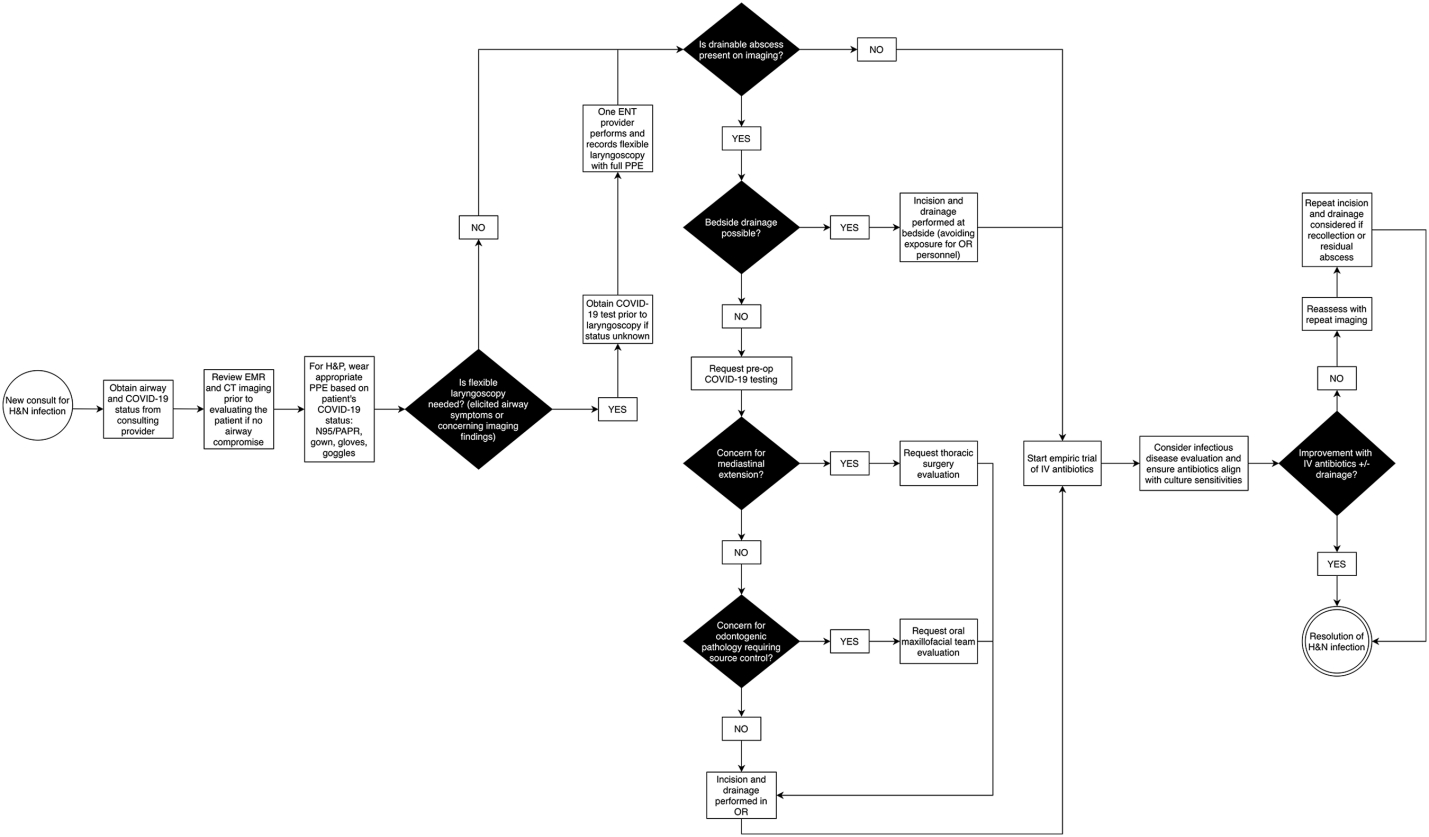
Algorithm for management of head and neck infection. Abbreviations: EMR, electronic medical record; PAPR, powered air purifying respirator; OR, operating room; CT, computed tomography; pre-op, pre-operative; IV, intravenous administration.

Once incision and drainage is performed, the patient will commonly have surgical drains, either an open system such as a Penrose or closed system such as Jackson-Pratt. In the case of an open drain system, it is advisable to apply gauze loosely at the open end of the drain to prevent contamination of the immediate surroundings (patient’s gown, bedsheets, etc.) by wound drainage. Some patients may have open wounds requiring dressing changes and debridement, which should be attempted at the bedside if possible with proper pre-procedure analgesia and appropriate PPE. If repeat surgical debridement is required, the total number of operative trips should be limited, and the procedure should be coordinated with other scheduled cases if possible. If the surgical drains yield a low output, consideration should be given to removing the drains before discharge, and the patient and any immediate caregivers should be instructed on proper wound care. This limits the need for home health care services, reducing the risk of additional exposure to healthcare workers and vice versa.

#### Otologic complaints (Figure 6)

Patients presenting to the emergency room with chief complaints such as otalgia, otorrhea, hearing loss, vertigo, and facial nerve dysfunction generally have an acute course or onset of symptoms. If a patient presents with chronic symptoms, one must assess for underlying cholesteatoma, malignancy, or chronic infection. Unless there is an acute change in symptomatology warranting inpatient admission, such patients may be discharged home with close follow-up as long as a definitive outpatient plan is established.

**Figure 6.**
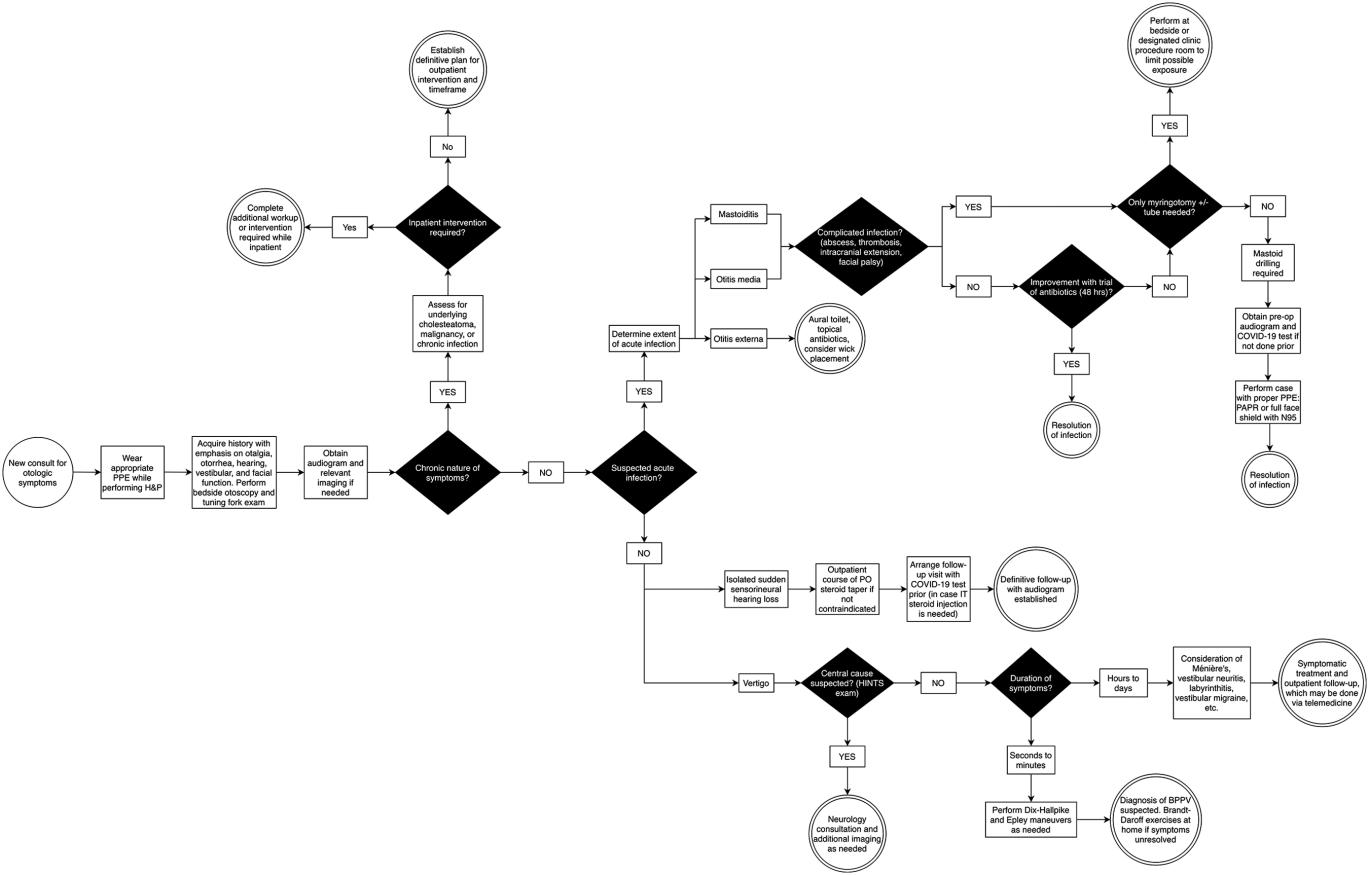
Algorithm for management of otologic symptoms. Abbreviations: PAPR, powered air purifying respirator; IT, intratympanic injection; HINTS, head impulse, nystagmus, and test of skew exam for vertigo; BPPV, benign paroxysmal positional vertigo.

For patients with an acute infection, it is imperative to determine if the infection is complicated; one should assess for the presence of an abscess, facial palsy, thrombosis, or intracranial involvement. In such cases, operative intervention is generally warranted in the manner of a myringotomy ± tube placement, cortical mastoidectomy, or a combination of both. If only myringotomy and tube insertion is required, it is recommended to perform a bedside procedure with a portable operative microscope or in a dedicated procedure room to avoid exposure to additional personnel in the OR. If a mastoidectomy is required, pre-operative COVID-19 testing should be requested along with a negative pressure room. The case is performed with full PPE as described above; a drape may be fashioned to reduce the bone dust droplet and aerosol spread.^25^ For uncomplicated infections, patients should first undergo a trial of antibiotics. If there is no improvement on an empiric antibiotic and physical exam findings are concerning, re-imaging may be warranted.

Patients presenting with an isolated, sudden sensorineural hearing loss are given an outpatient oral steroid course, and baseline audiogram is obtained. When arranging follow-up, it is advisable to obtain COVID-19 testing prior to clinic visit in the case that an intratympanic steroid injection is required.

#### Craniofacial trauma (Figure 7)

Craniofacial trauma consultations primarily occur in the ED. As with most ED consults, COVID-19 status is often unknown, so patients are treated as COVID-19 positive. For minor trauma in which clinical decisions can be made primarily based on imaging and history, an E-consult may be performed, and physical exam may be deferred to an outpatient follow up visit. This reduces the number of providers interacting with each patient in the acute setting. The decision for an E-consult is shared between the trauma and otolaryngology consult teams to ensure that patient care is not compromised.

**Figure 7.**
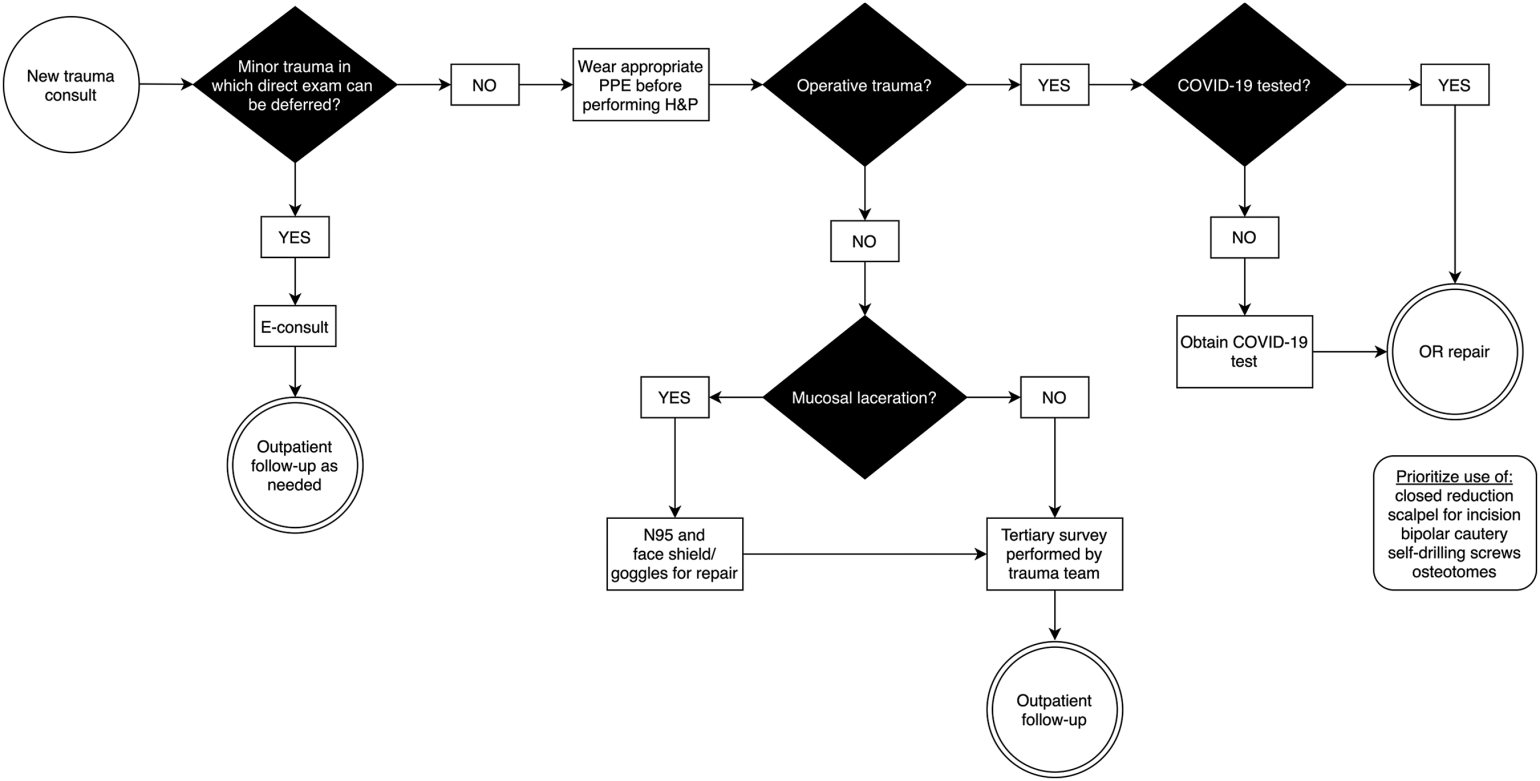
Algorithm for management of craniofacial trauma. Abbreviations: PPE, personal protective equipment; H&P, history and physical exam; E-consult, electronic consultation; OR, operating room.

Treatment of craniofacial trauma may require procedural intervention. Lacerations involving mucosal surfaces are considered high risk procedures,^26^ thus appropriate PPE is critical. Pre-procedural COVID-19 testing should be obtained if possible. Operative interventions range from low risk procedures that only require transcutaneous incisions to high risk procedures such as repair of mandible or nasal bone fractures that often require violation of mucosal surfaces or manipulation of the nasal cavity. The AO Foundation recommends additional strategies such as preference for closed reduction of fractures, use of scalpel over monopolar cautery for mucosal incisions, use of self-drilling screws, use of bipolar over monopolar cautery for hemostasis, and use of osteotomes over power saws for maxillofacial procedures.^27^

## Conclusion

Otolaryngology consultations provide valuable services ranging from evaluation of routine symptoms to critical airways. Increased risk of viral spread during the COVID-19 pandemic has been addressed by adapting common consult protocols for effective patient care with appropriate risk mitigation. Systematic otolaryngology consult service modifications are required in order to reduce risk of exposure while providing comprehensive patient care.
